# Supplementary material for: CRISPR-mediated gene correction links the ATP7A M1311V mutations with amyotrophic lateral sclerosis pathogenesis in one individual
Source: Commun Biol. 2020 Jan 20;3:33. doi: 10.1038/s42003-020-0755-1 (PMC6970999; doi:10.1038/s42003-020-0755-1)
Supplement: Supplementary file 2 — Description of Additional Supplementary Files [file 42003_2020_755_MOESM2_ESM.pdf]

Description of additional supplementary files

Supplementary Data 1. Source data used for graphs shown in Figure 3.

Supplementary Data 2. Source data used for graphs shown in Figure 4.
