# Supplementary material for: CRISPR-mediated gene correction links the ATP7A M1311V mutations with amyotrophic lateral sclerosis pathogenesis in one individual
Source: Commun Biol. 2020 Jan 20;3:33. doi: 10.1038/s42003-020-0755-1 (PMC6970999; doi:10.1038/s42003-020-0755-1)
Supplement: Supplementary file 4 — Supplementary Information [file 42003_2020_755_MOESM4_ESM.pdf]

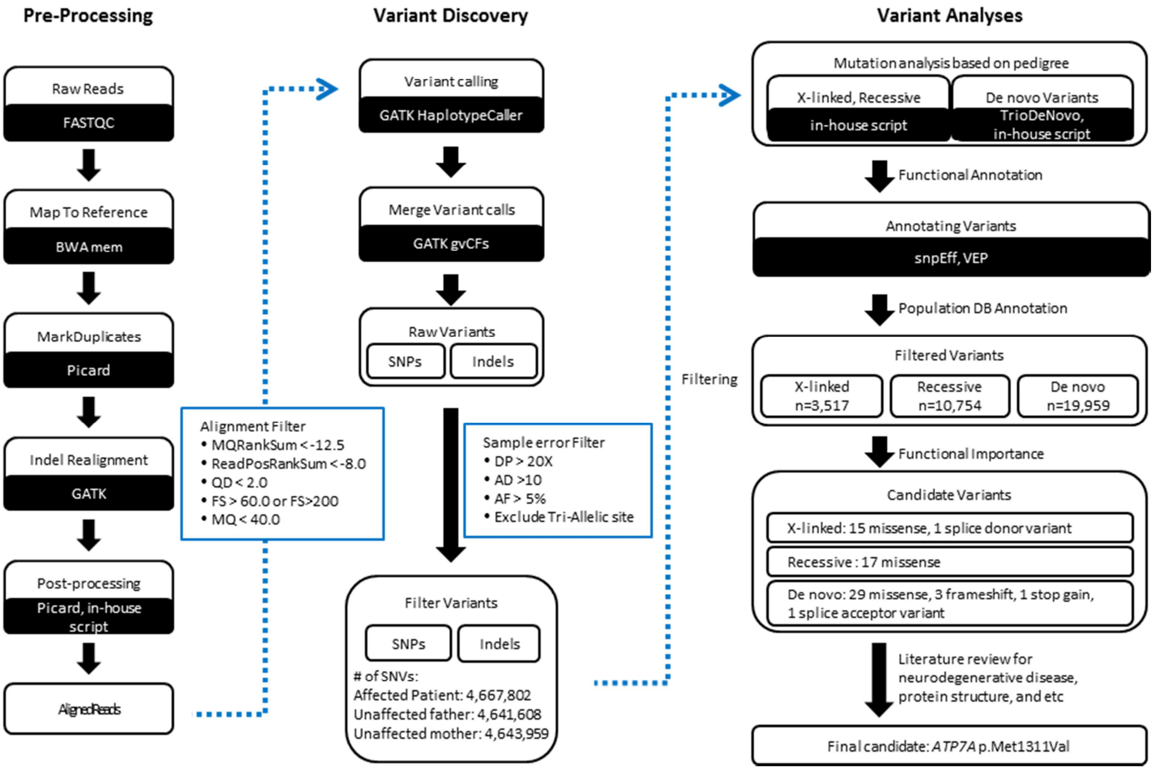

Supplementary Fig. 1 Schematic of the three main steps used to analyze trio WGS results: Pro-processing, Variant discovery, and Variant analyses. Raw sequences were aligned to the hg19 reference genome with BWA-mem. QC filtered alignment data, after duplication removal and indel realignment, were used for germline mutation calling using GATK HaplotypeCaller. Variants were categorized as inherited or *de novo* mutations, after which functional and population DB annotations were performed.

a

Symbol	CHROM	POS	REF	ALT	Protein variant	Nucleotide variant	Genetic Mode	Consequence
DTX1	12	113515320	AT	A	p.Trp118fs		De novo	frameshift_variant
	12	113515322	G	GAA	p.Trp118_Thr119fs		De novo	frameshift_variant+stop_gained
	12	113515335	T	G	p.Asp122Glu	c.366T>G	De novo	missense_variant
NOTCH2	1	120539711	G	T	p.Ser220Arg	c.660C>A	De novo	missense_variant
	1	120539668	T	A	p.Thr235Ser	c.703A>T	De novo	missense_variant
GFRA2	8	21560462	C	T	p.Gly253Asp	c.758G>A	De novo	missense_variant
GART	21	34877981	C	T	p.Arg871His	c.2612G>A	De novo	missense_variant
ATP7A	X	77298212	A	G	p.Met1311Val	c.3931A>G	X-linked	missense_variant

b

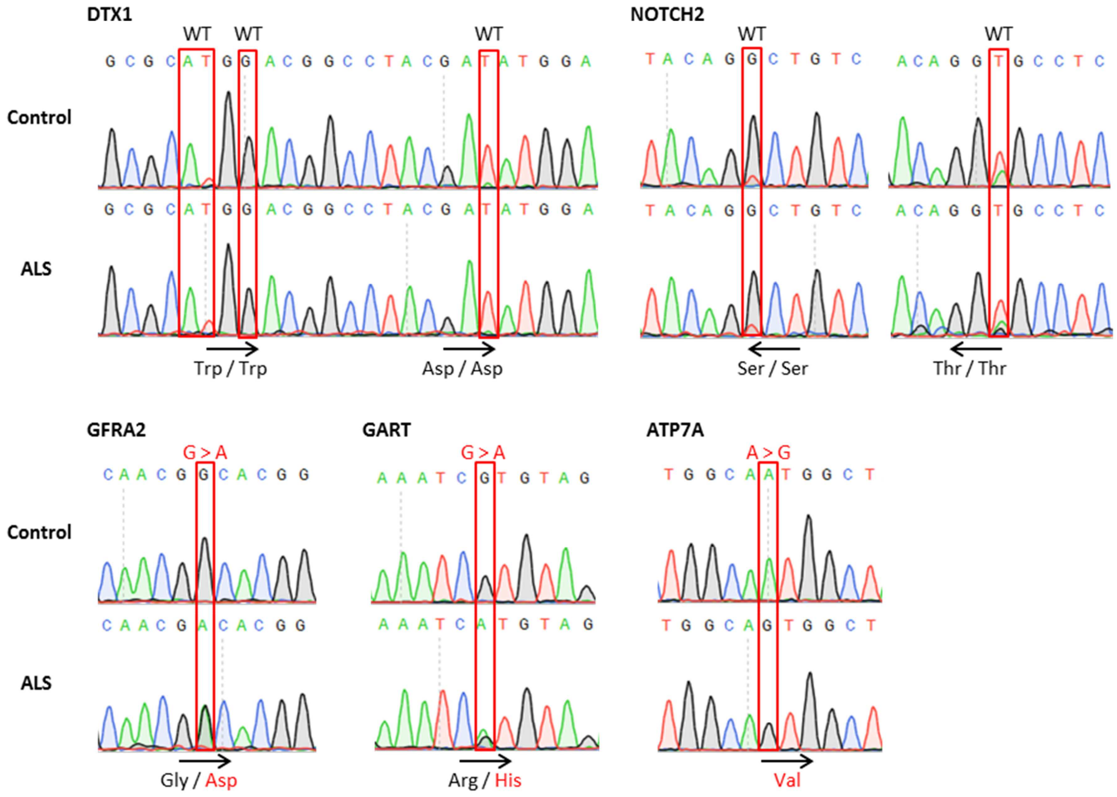

Supplementary Fig. 2 Confirmation of mutations by Sanger sequencing. **a,** Information of the predicted mutations in *DTX1*, *NOTCH2*, *GFRA2*, *GART* and *ATP7A* from the WGS. **b,** Sanger sequencing to confirm the predicted mutations in *DTX1*, *NOTCH2*, *GFRA2*, *GART*, and *ATP7A* in the ALS-patient derived fibroblasts and GM14867 as a negative control (Control, GM14867; ALS, the ALS-patient derived fibroblast). Each predicted mutation site is shown in a box. Variants in *DTX1* and *NOTCH2* are wild type, each mutation in *GFRA2* and *GART* is heterozygous and *ATP7A* is hemizygous. The predicted mutation sites and amino acids corresponding the sequencing results are indicated under the sequencing chromatogram (black, wild type; red, mutant). CHROM, Chromosome; POS, Position; REF, Reference; ALT, Alteration; WT, wild type.

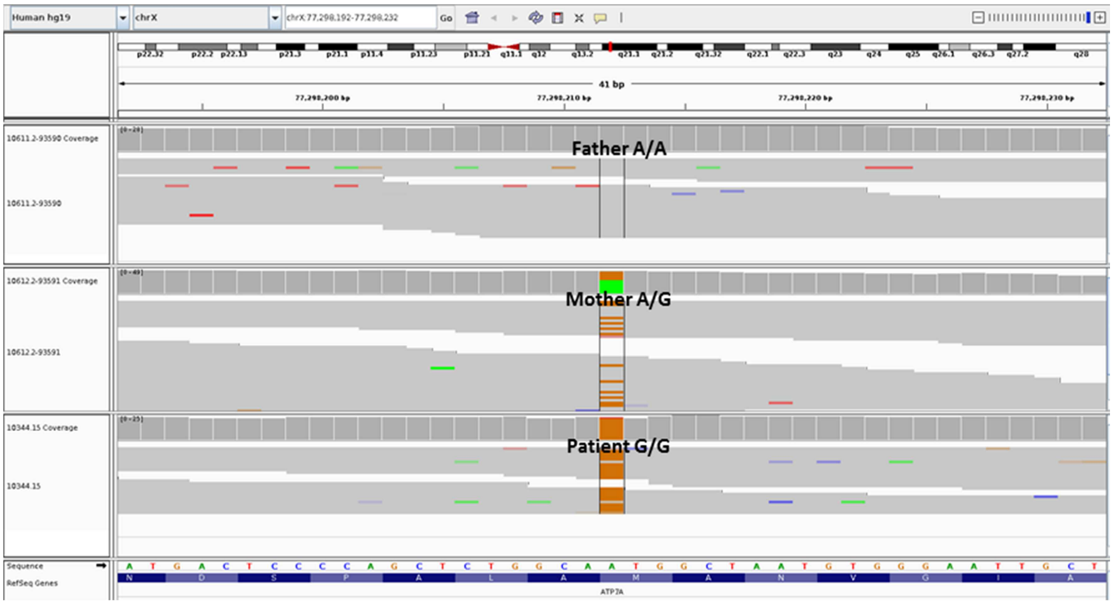

Supplementary Fig. 3 Visualizing ATP7A M1311V using Integrated Genomic Viewer. c.3931A>G was inherited by the male patient from the healthy, carrier mother. The patient is affected due to this X-linked recessive mutation.

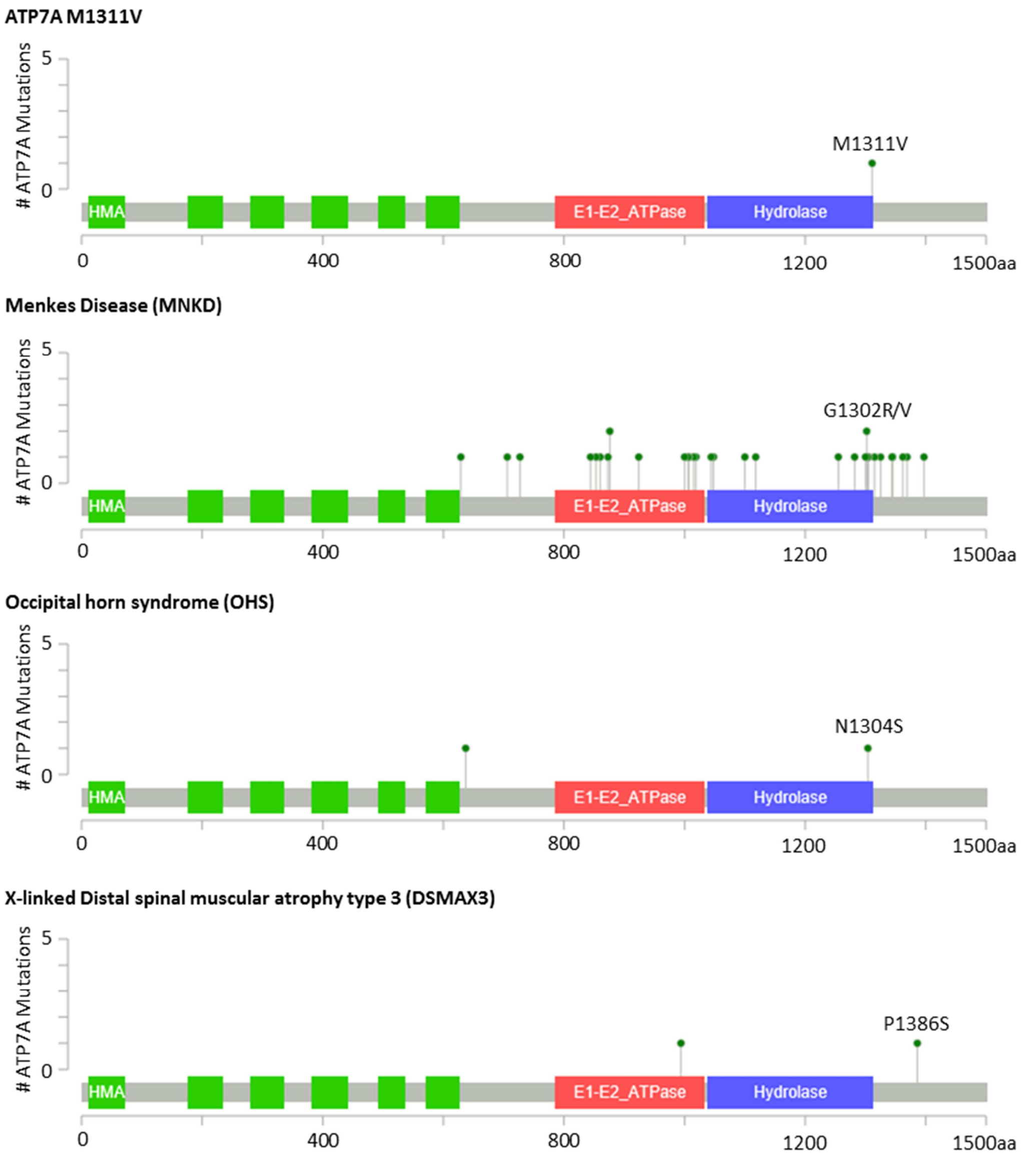

Supplementary Fig. 4 Locations of novel M1311V mutations and known causative mutations in *ATP7A* for Menkes disease, occipital horn syndrome, and X-linked distal spinal muscular atrophy type 3.

a

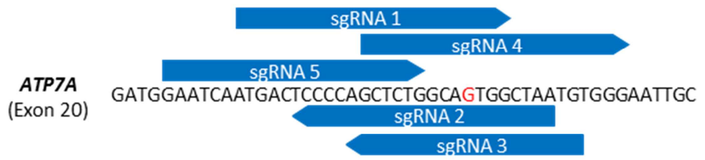

	Target (5' to 3')	Direction	GC Contents (%, w/o PAM)	Mismatches		
				0	1	2
sgRNA 1	TGACTCCCCAGCTCTGGCAGTGG	+	65	0	1	5
sgRNA 2	ATTAGCCACTGCCAGAGCTGGGG	-	55	0	1	2
sgRNA 3	ACATTAGCCACTGCCAGAGCTGG	-	55	0	1	2
sgRNA 4	GCTCTGGCAGTGGCTAATGTGGG	+	55	0	1	0
sgRNA 5	AATCAATGACTCCCCAGCTCTGG	+	50	1	0	1

b

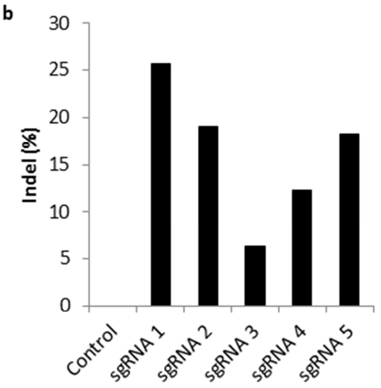

c

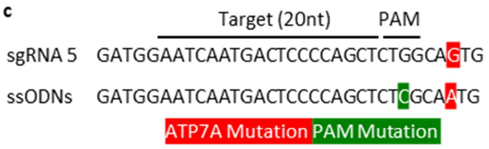

**ssODNs full sequence**	
sgRNA 5_ssODNs	TTCAATGGCTACATCTGTGCCTGTGCCAATAGCAATTCCCACATTAGCCATTGCGAGAGCTGGGGAGTCATTGATTCCATCTCCCACCATTGCTACCCGTTTCCC

d

**Deep sequencing primers**	
1st_Fwd	TGATAGATGCTGCCAGATGC
1st_Rev	GCTGGGTTGAGAAAGTCAGG
2nd_Fwd	ACACTCTTTCCCTACACGACGCTCTTCCGATCTTTGGCATTACTAAGGTGTTTGC
2nd_Rev	GTGACTGGAGTTCAGACGTGTGCTCTTCCGATCTGGGCAGTCTCATACTCCTCAA

Supplementary Fig. 5 CRISPR-Cas9 design for targeting ATP7A M1311V. **a,** 5 sgRNAs designed for gene correction in patient-derived iPS cells. **b,** Cleavage efficiency of the 5 sgRNAs. Formation of small insertions and deletions (indels) introduced by error prone nonhomologous end-joining was considered as evidence for Cas9-mediated cleavage. Based on these results, sgRNA 1 and 5 were chosen for *ATP7A* gene correction, and a gene-corrected cell line was established using sgRNA 5. **c,** The single-stranded oligodeoxynucleotides (ssODNs) used for establishing the ATP7A-Cor1 iPS cell line. The PAM sequence was disrupted by a synonymous mutation. **d,** Primers used for deep sequencing to confirm the *ATP7A* sequence after transfection of the ssODN and plasmids encoding Cas9 and the sgRNA.

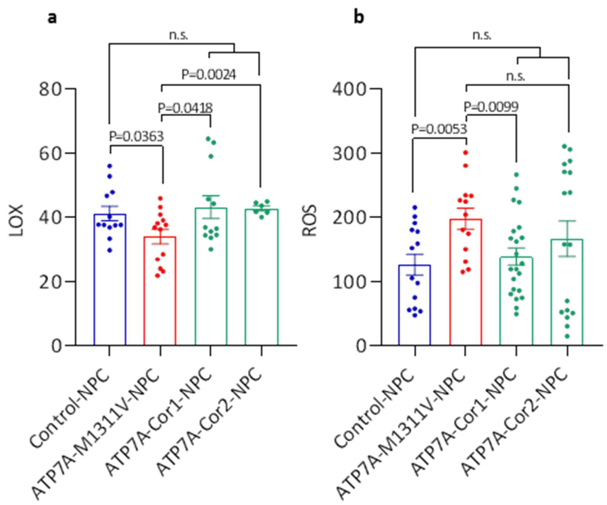

Supplementary Fig. 6 Improved LOX activity and ROS level in NPCs. **a,** Assessment of LOX activity in 2 isogenic NPC lines. n=12 (Control-NPC), n=13 (ATP7A-M1311V-NPC), n=12 (ATP7A-Cor1-NPC), and n=6 (ATP7A-Cor2-NPC). **b,** Quantification of ROS levels in 2 isogenic NPC lines. n=14 (Control-NPC), n=12 (ATP7A-M1311V-NPC), n=22 (ATP7A-Cor1-NPC), and n=17 (ATP7A-Cor2-NPC). Data are presented as mean ±s.e.m,; unpaired two-tailed t test with Welch's correction.

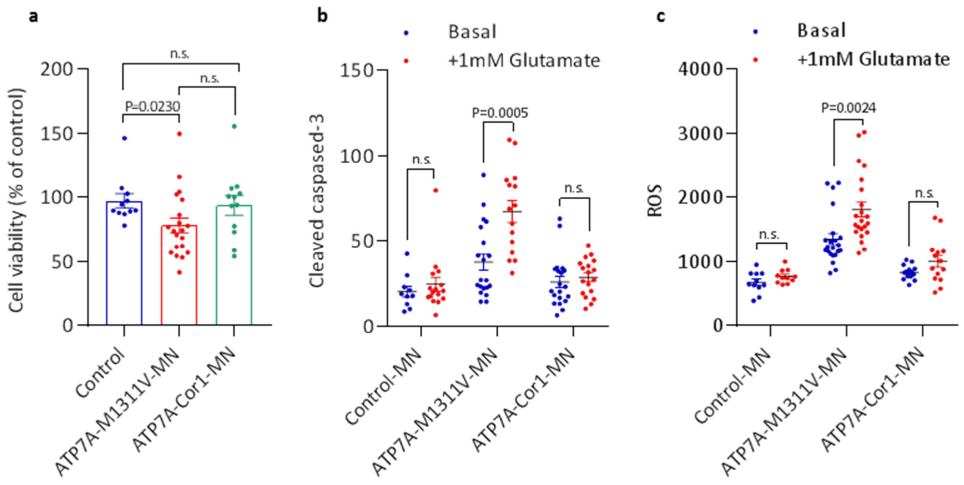

Supplementary Fig. 7 Glutamate vulnerability in MNs. **a,** Glutamate-induced cytotoxicity. Viability of MNs after treatment (1 mM glutamate for 1 hr) was compared to that of untreated cells. n=11 (Control-MN), n=20 (ATP7A-M1311V-MN), and n=12 (ATP7A-Cor1-MN). Data are presented as mean ±s.e.m,; unpaired two-tailed t test with Welch's correction. **b,** The cleaved caspase-3 fluorescence intensity was measured in MNs after 1 hr exposure to 1 mM glutamate. n=10 (Control-MN, basal), n=20 (ATP7A-M1311V-MN, basal), n=20 (ATP7A-Cor1-MN, basal), n=16 (Control-MN, 1 mM glutamate), n=15 (ATP7A-M1311V-MN, 1 mM glutamate), and n=18 (ATP7A-Cor1-MN, 1 mM glutamate). The error bars indicate ±s.e.m.; statistical analysis was performed using multiple t test with correction for multiple comparisons using the Holm-Sidak method. **c,** Quantification of ROS levels in MNs after 1 hr exposure to 1 mM glutamate. n=11 (Control-MN, basal), n=22 (ATP7A-M1311V-MN, basal), n=14 (ATP7A-Cor1-MN, basal), n=11 (Control-MN, 1 mM glutamate), n=22 (ATP7A-M1311V-MN, 1 mM glutamate), and n=14 (ATP7A-Cor1-MN, 1 mM glutamate). The error bars indicate ±s.e.m,; statistical analysis was performed using multiple t test with correction for multiple comparisons using the Holm-Sidak method.

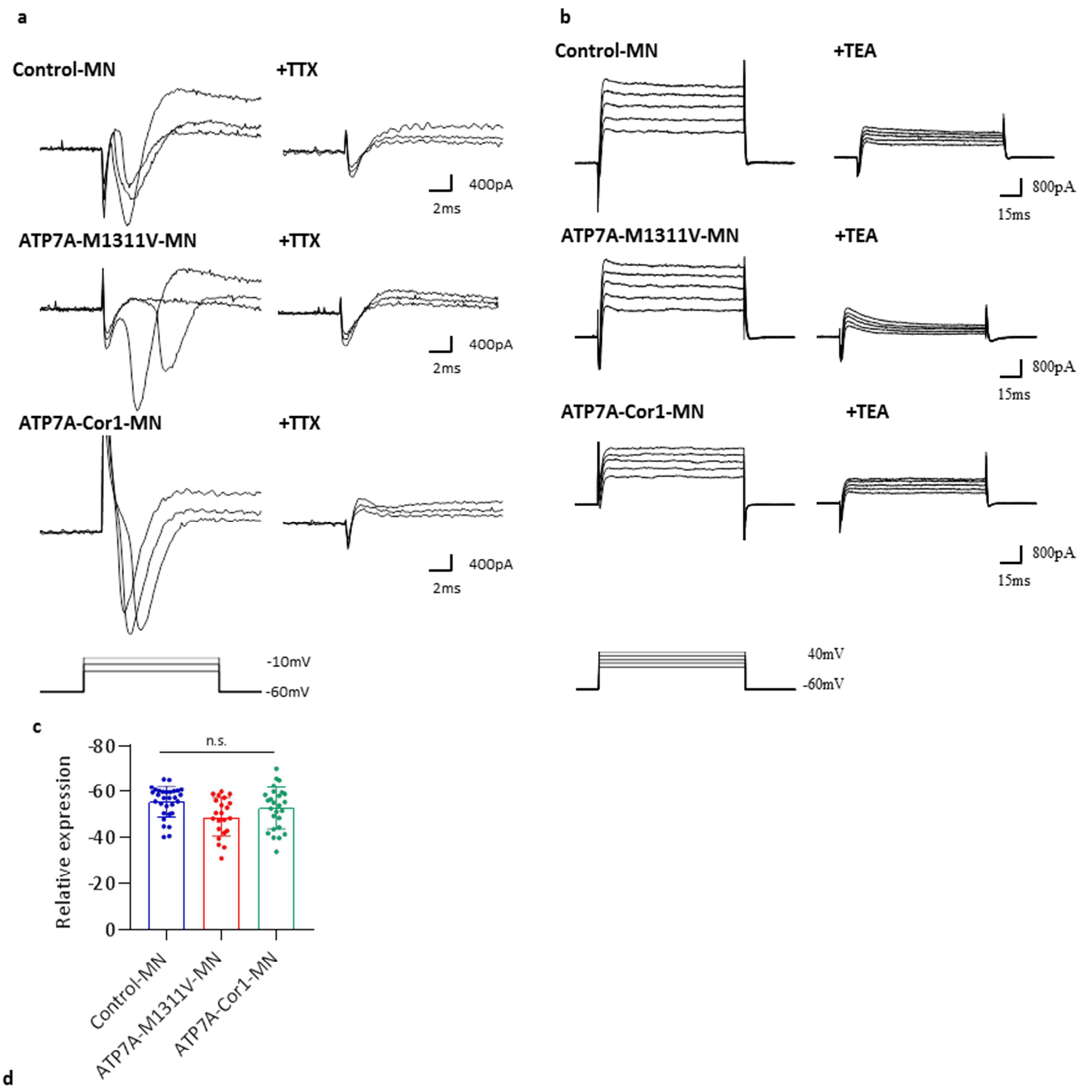

d

		Capacitance (pF)		Input Resistance (MΩ)		Series Resistance (MΩ)		RMP (mV)	
Group	n	Average	sem	Average	sem	Average	sem	Average	sem
Healthy donor	23	39.9735	5.78034	1696.76	180.621	15.7217	0.77086	-55.748	1.37356
ATP7A-M1311V	25	31.5	4.37	3299	486	13.9	0.69	-46	4.73
ATP7A-Cor1	30	29.8333	4.22077	2900.18	612.341	13.8667	0.71746	-49	3.90064

Supplementary Fig. 8 Current traces of voltage-gated sodium and potassium channels. **a,** Representative traces of sodium current from different cell types: Control-MN, ATP7A-M1311V-MN, and ATP7A-Cor1-MN. **b,** Representative traces of potassium currents from different cell types: Control-MN, ATP7A-M1311V-MN, and ATP7A-Cor1-MN. **c,** Similar values were observed for the resting membrane potential (RMP) for each cell type. The error bars indicate ±s.e.m.; statistical analysis was performed using two tailed t-test. *p<0.05, **p<0.01. **d,** Values for capacitance, input resistance, series resistance, and resting membrane potential (RMP).

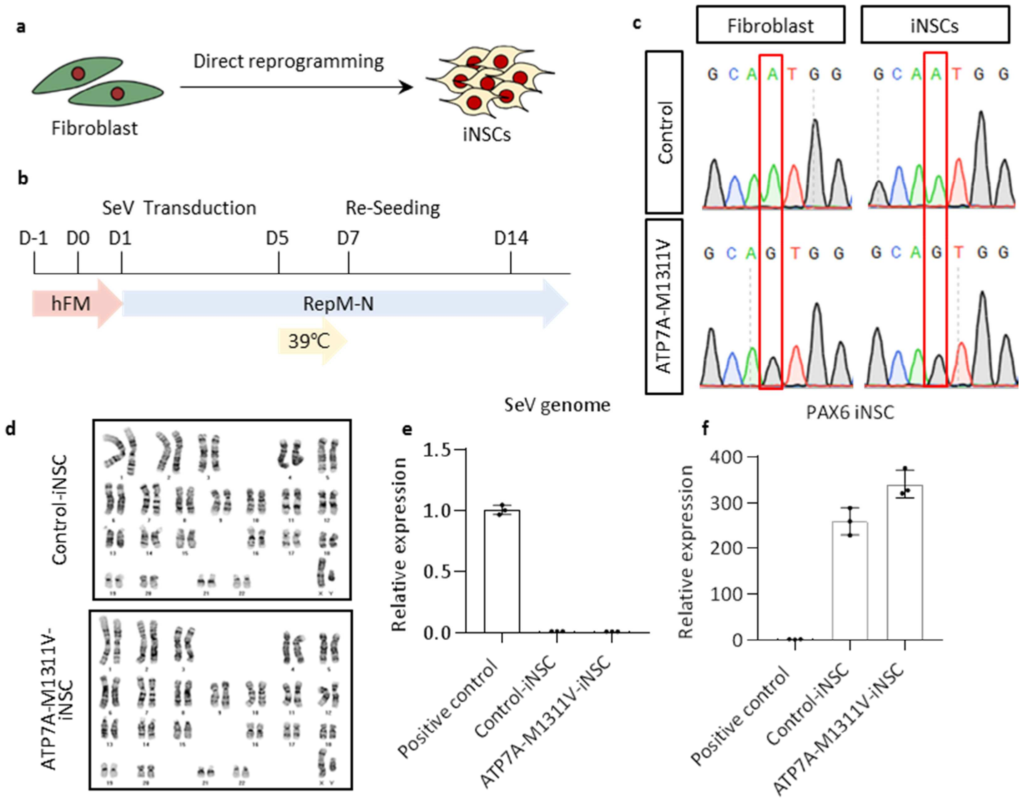
a
Direct reprogramming
Fibroblast
iNSCs
b
SeV Transduction
Re-Seeding
D-1
D0
D1
D5
D7
D14
hFM
RepM-N
39°C
c
Fibroblast
iNSCs
Control
ATP7A-M1311V
G C A A T G G
G C A G T G G
d
Control-iNSC
ATP7A-M1311V-iNSC
e
SeV genome
Relative expression
Positive control
Control-iNSC
ATP7A-M1311V-iNSC
f
PAX6 iNSC
Relative expression
Positive control
Control-iNSC
ATP7A-M1311V-iNSC

g

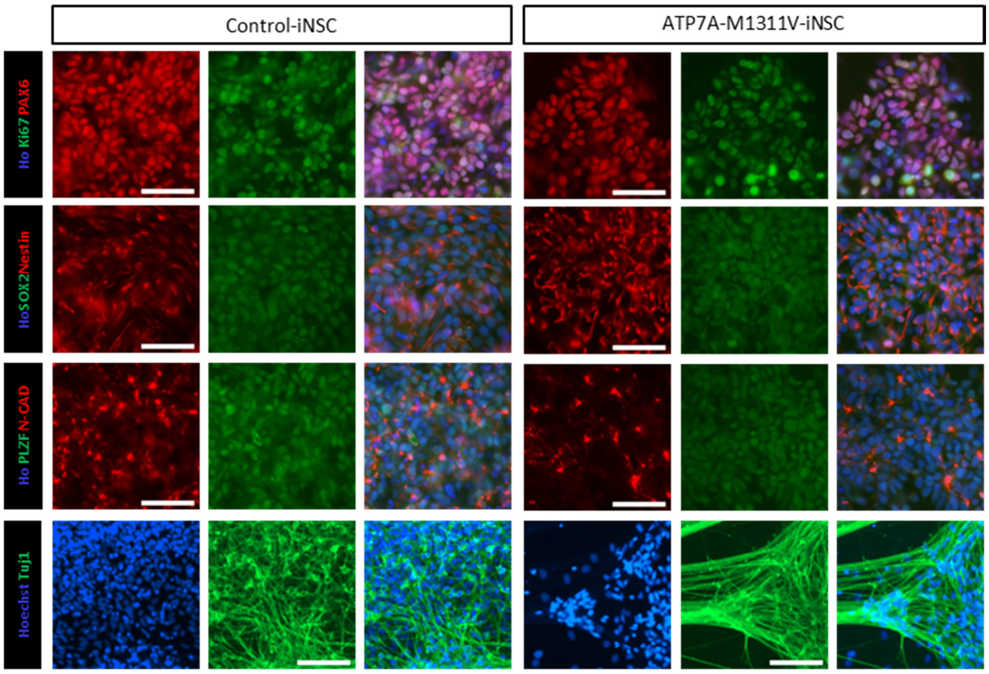

h

Locus/Clone	Control-fibroblast		Control-iNSC		ATP7A-M1311V-fibroblast		ATP7A-M1311V-iNSC	
D8S1179	12	13	12	13	10	14	10	14
D21S11	29	29.2	29	29.2	29	32.2	29	32.2
D7S820	11	13	11	13	8	12	8	12
CSF1PO	12	14	12	14	12	12	12	12
D3S1358	15	16	15	16	15	15	15	15
TH01	6	6	6	6	6	9.3	6	9.3
D13S317	10	14	10	14	8	12	8	12
D16S539	9	13	9	13	9	12	9	12
D2S1338	17	17	17	17	19	22	19	22
D19S433	13	14	13	14	12	13	12	13
vWA	15	16	15	16	16	18	16	18
TPOX	8	8	8	8	8	8	8	8
D18S51	12	15	12	15	13	13	13	13
D5S818	12	12	12	12	12	13	12	13
FGA	20	23	20	23	20	23	20	23
Gender	XY		XY		XY		XY	

Supplementary Fig. 9 Characterization of induced neural stem cells (iNSCs) directly reprogrammed from fibroblasts. **a,** Production of iNSCs from fibroblasts using direct reprogramming. **b,** Schematic representation of the iNSC generation process. SeV, Sendai virus. **c,** Sanger sequencing to confirm ATP7A M1311V and normal sequences in fibroblasts and iNSCs. **d,** Karyotype analysis of iNSCs from Control and patient. **e,** Q-PCR analysis of existing SeV genomes in iNSCs. Data are presented as mean ±s.d. **f,** Q-PCR analysis of neural stem cell marker PAX6. Data are presented as mean ±s.d. **g,** Immunostaining of iNSCs using antibodies against Ki67, PAX6, SOX2, Nestin, PLZF, and N-CAD; differentiated iNSCs using antibody against Tuj1. Hoechst was used for nuclear staining. Scale bars, 50 μm. **h,** Short tandem repeat analysis of fibroblasts and iNSCs from Control and patient.

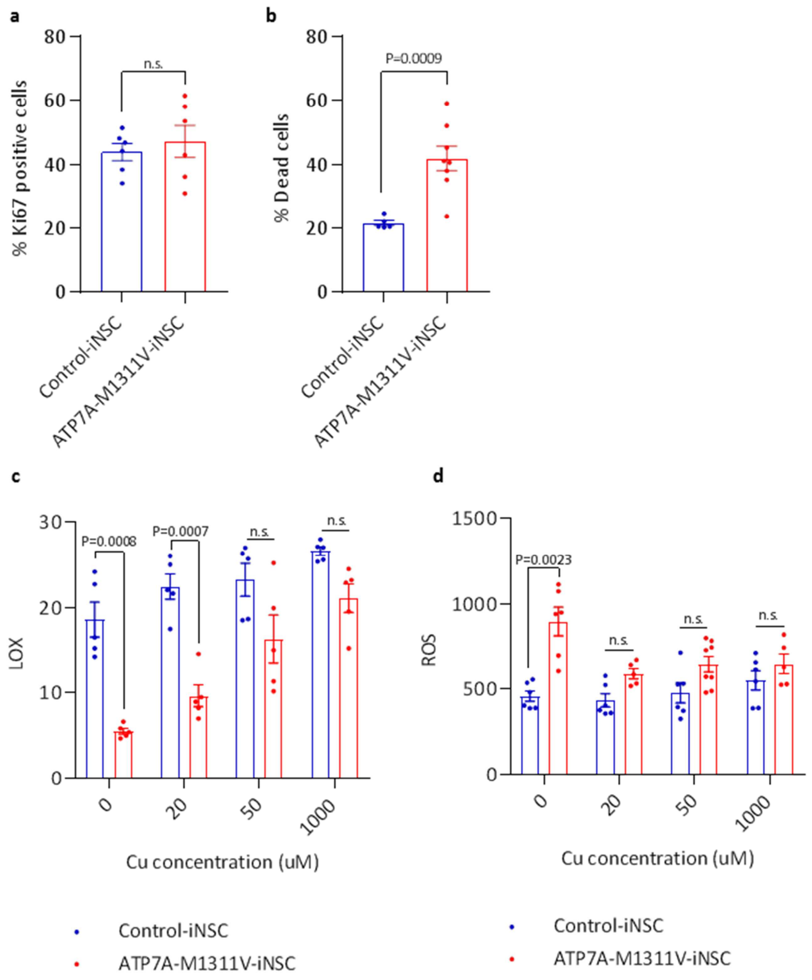

Supplementary Fig. 10 Examination of the functional differences between Control and patient-derived (ATP7A-M1311) iNSCs. **a,** The proportion of ki67 positive cells was not significantly different between Control-iNSC and ATP7A-M1311-iNSC cell populations. n=6. The error bars indicate ±s.e.m; statistical analysis was performed using unpaired two-tailed t test with Welch's correction. **b,** The proportion of dead cells was higher in the ATP7A-M1311 cell population. n=5 (Control-iNSC) and n=8 (ATP7A-M1311-iNSC). The error bars indicate ±s.e.m; statistical analysis was performed using unpaired two-tailed t test with Welch's correction. **c,** Comparison of LOX activity between Control-iNSC and ATP7A-M1311-iNSC cells. LOX activity was increased in response to exogenous Cu in ATP7A-M1311-iNSC cells. n=5. The error bars indicate ±s.e.m; statistical analysis was performed using multiple t test and Correct for multiple comparisons using the Bonferroni-Dunn method. **d,** Quantification of the ROS level. The ROS level decreased in response to exogenous Cu in ATP7A-M1311-iNSC cells; n=4 (0 µM Cu), n=5 (20 µM Cu), n=8 (50 µM Cu) and n=5 (100 µM Cu)compared to Control-iNSC cells; n=6. The error bars indicate ±s.e.m.; statistical analysis was performed using multiple t test and Correct for multiple comparisons using the Bonferroni-Dunn method.

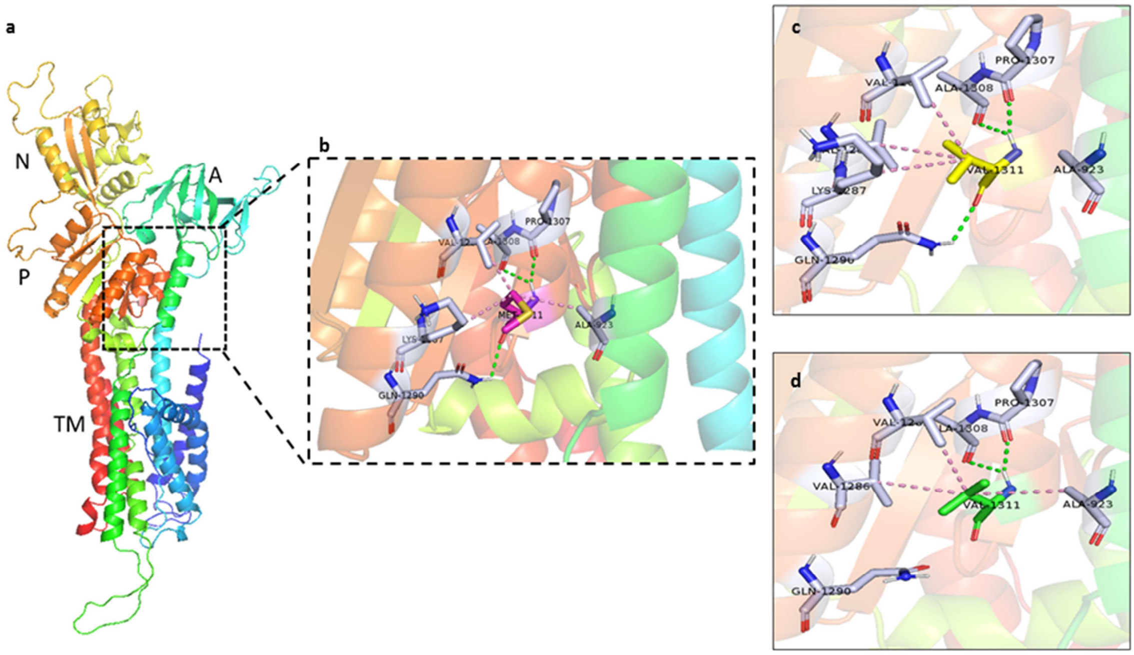

Supplementary Fig. 11 Homology model to show the effect of the M1311V mutation on ATP7A. **a,** The homology model consists of TM helices and three core (N-, P-, and A-) domains, shown in a cartoon representation. **b,** Residues interacting with methionine and valine are shown in a blue-white colored stick format. Met1311, shown in a magenta colored stick representation, is located in the P-domain. The P-domain helix is shown in orange. The α1 and α2 helices, which correspond to the A-domain, are shown in blue and green, respectively. Met1311 interacts with Gln1290, Pro1307, and Ala1308 through hydrogen bonds (green dashed lines) and also has hydrophobic contacts (light pink dashed lines) with Ala923, Val1283, and Lys1287. The mutated valine is shown in yellow and a green colored stick format in **c** and **d**, respectively. **c,** Depending on the orientation of the valine and surrounding residues, the M1311V mutation can break the hydrophobic contacts with Ala923, located in the A-domain α2 helix, or **d,** the hydrogen bond interaction with Gln1390.

Supplementary Table 1. High impact variants with allele frequencies < 0.001 in each genetic mode. CHROM, Chromosome; POS, Position; REF, Reference; ALT, Alteration; AF_ASJ, Allele frequency in the Ashkenazi Jewish population in the gnomAD database.

Symbol	CHROM	POS	REF	ALT	Protein variant	Genetic Mode	Consequence	AF_ASJ
NBPF1	1	16892272	C	CT	p.Ter975_Ser976fs	De novo	frameshift_variant	0
NOTCH2	1	120539711	G	T	p.Ser220Arg	De novo	missense_variant	0
NBPF8	1	144621649	C	G	p.Gln327Glu	De novo	missense_variant	0
SPDYE6	7	101993806	CG	C	p.Pro135fs	De novo	frameshift_variant	0
GFRA2	8	21560462	C	T	p.Gly253Asp	De novo	missense_variant	0
MUC19	12	40882660	C	A	p.Asn5302Lys	De novo	missense_variant	0
DTX1	12	113515335	T	G	p.Asp122Glu	De novo	missense_variant	0
KRTAP4-7	17	39240817	G	GTCGC	p.Cys120_Leu121fs	De novo	frameshift_variant	0
GART	21	34877981	C	T	p.Arg871His	De novo	missense_variant	0
GSTT2	22	24325648	C	T	p.Arg196*	De novo	stop_gained	0
ZBED1	X	2407148	G	A	p.Pro538Leu	X-linked	missense_variant	0
VCX3A	X	6451856	A	G	p.Leu164Pro	X-linked	missense_variant	0
VCX3A	X	6451872	C	G	p.Glu159Gln	X-linked	missense_variant	0
CXorf58	X	23933912	G	A		X-linked	splice_donor_variant	0
SUPT20HL2	X	24330930	C	T	p.Arg168His	X-linked	missense_variant	0
GLOD5	X	48624357	A	G	p.Met61Val	X-linked	missense_variant	0
BMP15	X	50654091	A	G	p.Asn103Ser	X-linked	missense_variant	0
DIAPH2	X	96171436	G	T		De novo	splice_acceptor_variant	0
ARMCX4	X	100749065	A	G	p.Glu1830Gly	X-linked	missense_variant	0
ARMCX4	X	100749077	A	G	p.Glu1834Gly	X-linked	missense_variant	0
ARMCX4	X	100749103	C	G	p.Pro1843Ala	X-linked	missense_variant	0

Supplementary Table 2. A list of ALS candidate genes with functionally important variants prioritized by a naïve Bayes algorithm. This algorithm propagates information to direct network neighbors. As a result, 7 genes were included in the nonsynonymous variants list of the patient (Candidate); these genes had not been previously characterized as ALS-related genes (Known). "Rank" represents a measure of the relevance of the gene to ALS in the prioritization list.

Rank	Ensembl_ID	Symbol	Known	Candidate
459	ENSG00000196126	HLA-DRB1	Unknown	After ASJ filter nonsynonymous
815	ENSG00000118873	RAB3GAP2	Unknown	After ASJ filter nonsynonymous
995	ENSG00000134250	NOTCH2	Unknown	After ASJ filter nonsynonymous
1185	ENSG00000154380	ENAH	Unknown	After ASJ filter nonsynonymous
1553	ENSG00000147202	DIAPH2	Unknown	After ASJ filter nonsynonymous
1558	ENSG00000162849	KIF26B	Unknown	After ASJ filter nonsynonymous
2358	ENSG00000145113	MUC4	Unknown	After ASJ filter nonsynonymous

Supplementary Table 3. A list of ALS candidate genes with functionally important variants prioritized by a Gaussian smoothing algorithm. This algorithm propagates information to all network neighbors. 47 genes were included in the nonsynonymous variants list of the patient (Candidate); these genes had not been previously characterized as ALS-related genes (Known). "Rank" represents a measure of the relevance of the gene to ALS in the prioritization list.

Rank	Ensembl_ID	Symbol	Known	Candidate
541	ENSG00000147202	DIAPH2	Unknown	After ASJ filter nonsynonymous
559	ENSG00000145113	MUC4	Unknown	After ASJ filter nonsynonymous
600	ENSG00000135144	DTX1	Unknown	After ASJ filter nonsynonymous
602	ENSG00000154380	ENAH	Unknown	After ASJ filter nonsynonymous
1025	ENSG00000134250	NOTCH2	Unknown	After ASJ filter nonsynonymous
1032	ENSG00000118873	RAB3GAP2	Unknown	After ASJ filter nonsynonymous
1600	ENSG00000162849	KIF26B	Unknown	After ASJ filter nonsynonymous
2196	ENSG00000143226	FCGR2A	Unknown	After ASJ filter nonsynonymous
2448	ENSG00000196126	HLA-DRB1	Unknown	After ASJ filter nonsynonymous
3440	ENSG00000013619	MAMLD1	Unknown	After ASJ filter nonsynonymous
3451	ENSG00000198331	HYLS1	Unknown	After ASJ filter nonsynonymous
3878	ENSG00000174371	EXO1	Unknown	After ASJ filter nonsynonymous
5141	ENSG00000154358	OBSCN	Unknown	After ASJ filter nonsynonymous
5145	ENSG00000196433	ASMT	Unknown	After ASJ filter nonsynonymous
5225	ENSG00000168546	GFRA2	Unknown	After ASJ filter nonsynonymous
5307	ENSG00000175221	MED16	Unknown	After ASJ filter nonsynonymous
5399	ENSG00000186432	KPNA4	Unknown	After ASJ filter nonsynonymous
6393	ENSG00000163541	SUCLG1	Unknown	After ASJ filter nonsynonymous
6432	ENSG00000086619	ERO1B	Unknown	After ASJ filter nonsynonymous
6918	ENSG00000111641	NOP2	Unknown	After ASJ filter nonsynonymous
7152	ENSG00000162909	CAPN2	Unknown	After ASJ filter nonsynonymous
7502	ENSG00000262919	CCNQ	Unknown	After ASJ filter nonsynonymous
8040	ENSG00000145864	GABRB2	Unknown	After ASJ filter nonsynonymous
8482	ENSG00000177535	OR2B11	Unknown	After ASJ filter nonsynonymous
8502	ENSG00000177186	OR2M7	Unknown	After ASJ filter nonsynonymous
8584	ENSG00000182783	OR2T29	Unknown	After ASJ filter nonsynonymous
8624	ENSG00000183269	OR52E8	Unknown	After ASJ filter nonsynonymous
8920	ENSG00000177201	OR2T12	Unknown	After ASJ filter nonsynonymous
9132	ENSG00000198753	PLXNB3	Unknown	After ASJ filter nonsynonymous
9464	ENSG00000143674	MAP3K21	Unknown	After ASJ filter nonsynonymous
9986	ENSG00000145439	CBR4	Unknown	After ASJ filter nonsynonymous
10153	ENSG00000160117	ANKLE1	Unknown	After ASJ filter nonsynonymous
10156	ENSG00000214717	ZBED1	Unknown	After ASJ filter nonsynonymous
10216	ENSG00000130385	BMP15	Unknown	After ASJ filter nonsynonymous
10231	ENSG00000128567	PODXL	Unknown	After ASJ filter nonsynonymous
10707	ENSG00000152591	DSPP	Unknown	After ASJ filter nonsynonymous
10953	ENSG00000177151	OR2T35	Unknown	After ASJ filter nonsynonymous
11922	ENSG00000116690	PRG4	Unknown	After ASJ filter nonsynonymous
12080	ENSG00000169059	VCX3A	Unknown	After ASJ filter nonsynonymous
12325	ENSG00000152104	PTPN14	Unknown	After ASJ filter nonsynonymous
12785	ENSG00000185420	SMYD3	Unknown	After ASJ filter nonsynonymous
13312	ENSG00000067840	PDZD4	Unknown	After ASJ filter nonsynonymous
13649	ENSG00000167384	ZNF180	Unknown	After ASJ filter nonsynonymous

14163	ENSG00000152779	SLC16A12	Unknown	After ASJ filter nonsynonymous
14184	ENSG00000219073	CELA3B	Unknown	After ASJ filter nonsynonymous
14388	ENSG00000079263	SP140	Unknown	After ASJ filter nonsynonymous
14464	ENSG00000133561	GIMAP6	Unknown	After ASJ filter nonsynonymous

Supplementary Table 4. 126 ALS-related genes for network-based prioritization.

Gene Symbol	Ensembl_ID	Major	GeneSymbol	Ensembl_ID	Major
AGT	ENSG00000135744		MAOB	ENSG00000069535	
ALAD	ENSG00000148218		MAPT	ENSG00000186868	
ALS2	ENSG00000003393	Major	MATR3	ENSG00000015479	Major
ALS3	N/A	Major	MT-ND2	ENSG00000198763	
ALS7	N/A	Major	NAIP	ENSG00000276068	
ANG	ENSG00000214274	Major	NEFH	ENSG00000100285	Major
APEX1	ENSG00000100823		NETO1	ENSG00000166342	
APOE	ENSG00000130203		NIPA1	ENSG00000170113	
AR	ENSG00000169083		NT5C1A	ENSG00000116981	
ARHGEF28	ENSG00000214944		OGG1	ENSG00000114026	
ATXN2	ENSG00000204842	Major	OMA1	ENSG00000162600	
B4GALT6	ENSG00000118276		OPTN	ENSG00000123240	Major
BCL11B	ENSG00000127152		PARK7	ENSG00000116288	
BCL6	ENSG00000113916		PCP4	ENSG00000183036	
C1orf27	ENSG00000157181		PFN1	ENSG00000108518	Major
C9orf72	ENSG00000147894	Major	PLEKHG5	ENSG00000171680	
CCS	ENSG00000173992		PON1	ENSG00000005421	
CDH13	ENSG00000140945		PON2	ENSG00000105854	
CDH22	ENSG00000149654		PON3	ENSG00000105852	
CHCHD10	ENSG00000250479	Major	PRPH	ENSG00000135406	Major
CHGB	ENSG00000089199		PSEN1	ENSG00000080815	
CHMP2B	ENSG00000083937	Major	PVR	ENSG00000073008	
CNTF	ENSG00000242689		RAMP3	ENSG00000122679	
CNTN4	ENSG00000144619		RBMS1	ENSG00000153250	
CNTN6	ENSG00000134115		RNASE2	ENSG00000169385	
CRIM1	ENSG00000277354		RNF19A	ENSG00000034677	
CRYM	ENSG00000103316		SARM1	ENSG00000004139	
CSNK1G3	ENSG00000151292		SCN7A	ENSG00000136546	
CST3	ENSG00000101439		SELL	ENSG00000188404	
CX3CR1	ENSG00000168329		SEMA6A	ENSG00000092421	
CYP2D6	ENSG00000272532		SETX	ENSG00000107290	Major
DAO	ENSG00000110887	Major	SIGMAR1	ENSG00000147955	Major
DCTN1	ENSG00000204843	Major	SLC1A2	ENSG00000110436	
DIAPH3	ENSG00000139734		SLC39A11	ENSG00000133195	
DISC1	ENSG00000162946		SMN1	ENSG00000275349	
DOC2B	ENSG00000272670		SMN2	ENSG00000273772	
DPP6	ENSG00000130226		SNCG	ENSG00000173267	
DYNC1H1	ENSG00000197102		SOD1	ENSG00000142168	Major
EFEMP1	ENSG00000115380		SOD2	ENSG00000112096	

ELP3	ENSG00000134014	Major	SOX5	ENSG00000134532	
EPHA4	ENSG00000116106		SPAST	ENSG00000021574	Major
ERBB4	ENSG00000178568	Major	SPG11	ENSG00000104133	Major
EWSR1	ENSG00000182944		SPG7	ENSG00000197912	
FEZF2	ENSG00000153266		SQSTM1	ENSG00000161011	Major
FGGY	ENSG00000172456		SS18L1	ENSG00000184402	
FIG4	ENSG00000112367	Major	SUSD1	ENSG00000106868	
FUS	ENSG00000089280	Major	SYNE	#N/A	
GARS	ENSG00000106105		SYT9	ENSG00000170743	
GLE1	ENSG00000119392		TAF15	ENSG00000276833	Major
GRB14	ENSG00000115290		TARDBP	ENSG00000120948	Major
GRN	ENSG00000030582		TBK1	ENSG00000183735	
HEXA	ENSG00000213614		TRPM7	ENSG00000092439	
HFE	ENSG00000010704		TUBA4A	ENSG00000127824	
HNRNPA1	ENSG00000135486	Major	UBQLN2	ENSG00000188021	Major
HNRNPA2B1	ENSG00000122566		UNC13A	ENSG00000130477	Major
ITPR2	ENSG00000123104		VAPB	ENSG00000124164	Major
KDR	ENSG00000128052		VCP	ENSG00000165280	Major
KIFAP3	ENSG00000075945		VDR	ENSG00000111424	
LIF	ENSG00000128342		VEGFA	ENSG00000112715	
LIPC	ENSG00000166035		VPS54	ENSG00000143952	
LMNB1	ENSG00000113368	Major	ZFP64	ENSG00000020256	
LOX	ENSG00000113083		ZNF512B	ENSG00000196700	
LUM	ENSG00000139329		ZNF746	ENSG00000181220	

Supplementary Table 5. Functional annotation using the *in silico* analysis tools SIFT and PolyPhen-2. In SIFT, 0 indicates "damaging" and 1 indicates "tolerated." In PolyPhen-2, 0 indicates "benign" and 1 indicates "pathogenic." Variants in *ATP7A* and *GART* were determined to be the most pathogenic. However, *GART* was eliminated as a candidate by literature review. CHROM, Chromosome; POS, Position; REF, Reference; ALT, Alteration.

Variant Types	CHROM	POS	REF	ALT	Symbol	SIFT	PolyPhen-2
X-linked	X	13799039	C	T	GPM6B	deleterious(0.03)	possibly damaging(0.717)
X-linked	X	49454026	T	C	PAGE1	deleterious(0)	possibly damaging(0.728)
X-linked	X	50658966	G	A	BMP15	tolerated(0.33)	benign(0.014)
X-linked	X	54836239	A	G	MAGED2	tolerated low confidence(0.14)	benign(0.04)
X-linked	X	77298212	A	G	ATP7A	deleterious(0.01)	probably damaging(0.965)
X-linked	X	130678349	C	T	OR13H1	tolerated(1)	benign(0.001)
Recessive	1	22310807	A	G	CELA3B	tolerated(1)	benign(0.014)
Recessive	1	170521376	G	A	GORAB	deleterious low confidence(0.01)	possibly damaging(0.769)
Recessive	1	248457979	C	A	OR2T12	deleterious(0)	benign(0.092)
Recessive	1	248458717	C	G	OR2T12	tolerated(1)	benign(0)
Recessive	19	55086249	T	C	LILRA2	tolerated(1)	benign(0.001)
Recessive	3	73111809	A	G	EBLN2	tolerated(1)	benign(0)
Recessive	3	97868419	G	A	OR5H14	deleterious(0.03)	benign(0.12)
Recessive	3	97888042	A	T	OR5H15	deleterious(0.03)	benign(0.158)
Recessive	4	69681936	G	T	UGT2B10	deleterious(0.01)	possibly damaging(0.546)
Recessive	6	31322911	C	T	HLA-B	tolerated low confidence(0.05)	probably damaging(0.991)
Recessive	6	31324892	G	C	HLA-B	tolerated low confidence(1)	benign(0.001)
Recessive	6	31324895	G	C	HLA-B	deleterious low confidence(0.04)	probably damaging(0.997)
Recessive	6	32726803	C	T	HLA-DQB2	tolerated(0.08)	benign(0.064)
Recessive	6	150209717	C	G	RAET1E	deleterious(0.05)	benign(0.042)
Recessive	7	47925331	C	G	PKD1L1	tolerated(1)	benign(0)
Recessive	7	94946084	A	T	PON1	deleterious(0)	benign(0.076)
Recessive	7	138603282	T	C	KIAA1549	tolerated(0.61)	benign(0.012)
De novo	1	17085791	G	A	MST1L	damaging(0)	probably damaging(0.89917)
De novo	1	115053498	A	G	TRIM33	tolerated(0.53)	benign(0.005)
De novo	1	120539668	T	A	NOTCH2	tolerated(0.15)	probably damaging(0.998)
De novo	1	120539711	G	T	NOTCH2	tolerated(0.46)	probably damaging(1.0)
De novo	1	161487805	T	C	FCGR2A	tolerated(0.11)	benign(0)
De novo	2	84676864	C	G	SUCLG1	tolerated(0.32)	benign(0)
De novo	4	338130	C	G	ZNF141	damaging(0.04)	possibly damaging(0.44986)
De novo	6	32489891	C	T	HLA-DRB5	damaging(0.02)	benign(0.19331)
De novo	6	32548627	C	T	HLA-DRB1	tolerated(0.36)	benign(0.14012)
De novo	7	131195073	G	A	PODXL	tolerated(0.15)	possibly damaging(0.47604)
De novo	8	21560462	C	T	GFRA2	tolerated(0.06)	probably damaging(0.998)
De novo	10	51827896	C	T	FAM21A	tolerated(0.23)	benign(0.012)
De novo	11	18267478	G	A	SAA2	tolerated(0.66)	benign(0.26081)
De novo	12	113515335	T	G	DTX1	tolerated(0.78)	probably damaging(0.986)
De novo	15	89400043	G	C	ACAN	tolerated(1)	benign(0.11235)
De novo	17	39240804	C	A	KRTAP4-7	tolerated(0.64)	benign(0.33748)
De novo	17	39240805	G	T	KRTAP4-7	tolerated(0.3)	benign(0.18461)
De novo	17	39240807	C	T	KRTAP4-7	tolerated(0.23)	benign(0.06786)
De novo	17	39240810	T	A	KRTAP4-7	tolerated(1)	benign(0.06786)

De novo	17	39274435	A	T	KRTAP4-11	tolerated(0.07)	possibly damaging(0.37525)
De novo	17	39274437	C	T	KRTAP4-11	damaging(0.03)	possibly damaging(0.46395)
De novo	19	875264	C	T	MED16	tolerated(0.46)	probably damaging(0.997)
De novo	21	34877981	C	T	GART	damaging(0)	probably damaging(1.0)

Supplementary Table 6. Additional gene information.

Symbol	Description	Consequence	Related disease	Related pathway	Location
DIAPH2	Diaphanous Related Formin 2	Splice_acceptor	Premature Ovarian Failure 2A Premature Ovarian Failure 1	Signaling by Rho GTPases Regulation of actin cytoskeleton.	(Splice_acceptor variant)
BMP15	Bone Morphogenetic Protein 15	Missense	Ovarian Dysgenesis 2 46,Xx Sex Reversal	SMAD Signaling Network PEDF Induced Signaling.	Non-functional domain
ZBED1	Zinc Finger BED-Type Containing 1	Missense	Laryngostenosis	NA	Non-functional domain
VCX3A	Variable Charge X-Linked 3A	Missense	Ichthyosis, X-Linked Opitz-Kaveggia Syndrome	NA	Non-functional domain

Supplementary Table 7. Off-target analysis in gene-corrected isogenic iPS cell lines. **a,** Potential off-target sites that had 1~3-nt mismatches or 1-nt DNA or RNA bulges were selected using Cas-OFFinder (http://www.rgenome.net/cas-offinder/). Additionally, one off-target site bearing 4-nt mismatches in the PAM-distal region was chosen.

	Bulge Type	Target	Chr	Position	Direction	Mismatches	Bulge size
off_1	RNA	AcTCAAT-ACTCCCCAGCTCAGG	12	1660448	-	1	1
off_2	RNA	AATCAATGACT-CCCAcCTCAGG	16	56852591	+	1	1
		AATCAATGACTC-CCAcCTCAGG			+	1	1
		AATCAATGACTCC-CAcCTCAGG			+	1	1
		AATCAATGACTCCC-AcCTCAGG			+	1	1
off_3	X	gATCAATGACTtCCCAGCTtAGG	12	94708925	+	3	0
off_4	X	cATCAcTGACTtCCCAGCTCCGG	3	140914524	+	3	0
off_5	X	AATCAATGACTCtCaAGCgCTGG	4	84241795	-	3	0
off_6	X	AATCAAaGACTtCCCAGCcCCGG	19	29775743	+	3	0
off_7	X	AATCAgTGACTCCaCAGaTCTGG	19	50830051	-	3	0
off_8	X	AATCAATGACTgCCCAGgTCAGG	10	10681368	+	2	0
off_9	X	AAcCcATGACTCCCaAGCTCTGG	6	41372500	-	3	0
off_10	X	AAcCAATGACTCCCCAGaTaGGG	6	135877917	-	3	0
off_11	X	AtTCAATGAaTCCCaAGCTCTGG	9	76707110	+	3	0
off_12	X	AATCAATcAtTCCCCAtCTCTGG	9	107375539	+	3	0
off_13	X	AgTCAgTGAaTCCCCAGCTCAGG	11	114559257	+	3	0
off_14	X	tAcCcgTGACTCCCCAGCTCAGG	16	12576772	+	4	0

Supplementary Table 8. Indel frequencies at the potential off-target sites. Indel frequencies at off_2 were not determined, and we reasoned that the sequencing procedure might have been interrupted by the T repeat (23bp) in the target region.

	**Indel (%)**													
	off_1	off_2	off_3	off_4	off_5	off_6	off_7	off_8	off_9	off_10	off_11	off_12	off_13	off_14
Patient iPS	0	ND	0	0	0	0	0	0.1	0	0	0	0	0	0
ATP7A-M1311V	0		0.1	0	0	0	0	0.1	0	0	0	0	0	0
ATP7A-Cor1	0		0.1	0.1	0	0.1	0	0.1	0	0	0	0	0	0
ATP7A-Cor2	0.1		0.1	0	0	0.1	0	0.1	0	0	0	0	0	0

Supplementary Table 9. Primers used for off-target analysis. Chr, Chromosome.

ATP7A_off_1_1stF	GGAAGGCACAGTTCTCAGGA
ATP7A_off_1_1stR	CCAGGATCCACTAAGGCAAG
ATP7A_off_1_2ndF	ACACTCTTTCCCTACACGAC GCTCTTCCGATCT AAACACTTGGGCACACACAG
ATP7A_off_1_2ndR	GTGACTGGAGTTCAGACGTGT GCTCTTCCGATCT TGTTCTGGCTGTGAGTGAATTT
ATP7A_off_2_1stF	GAGGATGGGAAGAGGGAAAG
ATP7A_off_2_1stR	GGAAGAGGCCCCTTATTGTC
ATP7A_off_2_2ndF	ACACTCTTTCCCTACACGAC GCTCTTCCGATCT AGTCTCCAAGGCTGTAGGCA
ATP7A_off_2_2ndR	GTGACTGGAGTTCAGACGTGT GCTCTTCCGATCT TTCTCCACTTGGCAGTTCCT
ATP7A_off_3_1stF	CACCATCACAGCAATCCAGT
ATP7A_off_3_1stR	GTAAGCCAGTCCCATCCGTA
ATP7A_off_3_2ndF	ACACTCTTTCCCTACACGAC GCTCTTCCGATCT TGCTGGGAAAATGTCATTAGG
ATP7A_off_3_2ndR	GTGACTGGAGTTCAGACGTGT GCTCTTCCGATCT GGGATTTTGAGGCCCTCTC
ATP7A_off_4_1stF	CCTGCAATCTTAAGGCCAAC
ATP7A_off_4_1stR	TACACGATTGGTTCATTTGTGC
ATP7A_off_4_2ndF	ACACTCTTTCCCTACACGAC GCTCTTCCGATCT GAAGCATCAGTTCTAACCATG
ATP7A_off_4_2ndR	GTGACTGGAGTTCAGACGTGT GCTCTTCCGATCT TCCATTGATTGCTTCCAAGAG
ATP7A_off_5_1stF	AGGAAGGGGCCAGACTTGTA
ATP7A_off_5_1stR	AGAGCCACCCCATTGAAGAT
ATP7A_off_5_2ndF	ACACTCTTTCCCTACACGAC GCTCTTCCGATCT TGAGAATCAGGCACTGGGG
ATP7A_off_5_2ndR	GTGACTGGAGTTCAGACGTGT GCTCTTCCGATCT CTAACAACTGTCAGGTGATGAA
ATP7A_off_6_1stF	TTGCTCTCTGCGTGCTCATA
ATP7A_off_6_1stR	ACAGTGCCCACCTTTCTCCT
ATP7A_off_6_2ndF	ACACTCTTTCCCTACACGAC GCTCTTCCGATCT GGGCGATTCCCCATTAACAAT
ATP7A_off_6_2ndR	GTGACTGGAGTTCAGACGTGT GCTCTTCCGATCT GCCTGTCTGGGGAGAGATTT
ATP7A_off_7_1stF	GGAGGCAGAGACAGAATTGC
ATP7A_off_7_1stR	TTGTTAAGCCCCTTGTCCAC
ATP7A_off_7_2ndF	ACACTCTTTCCCTACACGAC GCTCTTCCGATCT GATGTGGACTTGCTGTCTGG
ATP7A_off_7_2ndR	GTGACTGGAGTTCAGACGTGT GCTCTTCCGATCT CGTGTGCAGTGGGTATACAAG
ATP7A_off_8_1stF	AGCCTAGGACTTCCAGCACA
ATP7A_off_8_1stR	TGAATTAGGAGCTGGGAGGA
ATP7A_off_8_2ndF	ACACTCTTTCCCTACACGAC GCTCTTCCGATCT TCACCTACCTTATTGATCAGCG
ATP7A_off_8_2ndR	GTGACTGGAGTTCAGACGTGT GCTCTTCCGATCT AGTCTGTGGCTCCTTCCACT
ATP7A_off_9_1stF	CTGCAGAGTGGAACGACCTT
ATP7A_off_9_1stR	GCTTTGCTCCTTGAGTTGCT
ATP7A_off_9_2ndF	ACACTCTTTCCCTACACGAC GCTCTTCCGATCT GCTGTGGACGCTCAAAGAAT
ATP7A_off_9_2ndR	GTGACTGGAGTTCAGACGTGT GCTCTTCCGATCT TGCCAAGTGCTTAGGAGACG
ATP7A_off_10_1stF	TCACTTCCCTGGAATGATGAC
ATP7A_off_10_1stR	TCTCTGTCCATACCCCTTGG
ATP7A_off_10_2ndF	ACACTCTTTCCCTACACGAC GCTCTTCCGATCT TTAAGCCAGCAACGTCAGTG
ATP7A_off_10_2ndR	GTGACTGGAGTTCAGACGTGT GCTCTTCCGATCT GCTTCCTTTCCCCAGAACTC
ATP7A_off_11_1stF	GGGCTCAATATCACCCTGAA
ATP7A_off_11_1stR	CAAGGCAACTGGAGTGAACA
ATP7A_off_11_2ndF	ACACTCTTTCCCTACACGAC GCTCTTCCGATCT TGCTGGCTCTGAGCTGTCTA
ATP7A_off_11_2ndR	GTGACTGGAGTTCAGACGTGT GCTCTTCCGATCT GATGCCAGGGTCATTTCAAC
ATP7A_off_12_1stF	GCAATGTGGAGGTCGCTAGT
ATP7A_off_12_1stR	GCAAACCCAGACAGAGGTGT
ATP7A_off_12_2ndF	ACACTCTTTCCCTACACGAC GCTCTTCCGATCT TCACGTAGGTGGGAGAGAGAA
ATP7A_off_12_2ndR	GTGACTGGAGTTCAGACGTGT GCTCTTCCGATCT AATAGACACATGGCCCCAAC
ATP7A_off_13_1stF	GCCCTATGTCCAAGCAATTC
ATP7A_off_13_1stR	TGTCTGCTGAGGGGTTCTCT
ATP7A_off_13_2ndF	ACACTCTTTCCCTACACGAC GCTCTTCCGATCT GAATCCTTCCTGCTTTTCTGG
ATP7A_off_13_2ndR	GTGACTGGAGTTCAGACGTGT GCTCTTCCGATCT GTATGACATTGTTTGGGTGGAA
ATP7A_off_14_1stF	CTGCTCAGACGAACTGCATC
ATP7A_off_14_1stR	CACATCATGAGAGGCAATGG
ATP7A_off_14_2ndF	ACACTCTTTCCCTACACGAC GCTCTTCCGATCT GGACCCAACTCCCAAACCTA
ATP7A_off_14_2ndR	GTGACTGGAGTTCAGACGTGT GCTCTTCCGATCT AATAGGAACCTGGTCAATTACG
