# Supplementary material for: CRISPR-mediated gene correction links the ATP7A M1311V mutations with amyotrophic lateral sclerosis pathogenesis in one individual
Source: Commun Biol. 2020 Jan 20;3:33. doi: 10.1038/s42003-020-0755-1 (PMC6970999; doi:10.1038/s42003-020-0755-1)
Supplement: Supplementary file 5 — Reporting Summary [file 42003_2020_755_MOESM5_ESM.pdf]

nature research

Corresponding author(s): Sangsu Bae

Last updated by author(s): Dec 10, 2019

Reporting Summary

Nature Research wishes to improve the reproducibility of the work that we publish. This form provides structure for consistency and transparency in reporting. For further information on Nature Research policies, see Authors & Referees and the Editorial Policy Checklist.

Statistics

For all statistical analyses, confirm that the following items are present in the figure legend, table legend, main text, or Methods section.

n/a	Confirmed	
☐	☒	The exact sample size (n) for each experimental group/condition, given as a discrete number and unit of measurement
☐	☒	A statement on whether measurements were taken from distinct samples or whether the same sample was measured repeatedly
☐	☒	The statistical test(s) used AND whether they are one- or two-sided<br>*Only common tests should be described solely by name; describe more complex techniques in the Methods section.*
☒	☐	A description of all covariates tested
☒	☐	A description of any assumptions or corrections, such as tests of normality and adjustment for multiple comparisons
☐	☒	A full description of the statistical parameters including central tendency (e.g. means) or other basic estimates (e.g. regression coefficient) AND variation (e.g. standard deviation) or associated estimates of uncertainty (e.g. confidence intervals)
☐	☒	For null hypothesis testing, the test statistic (e.g. F, t, r) with confidence intervals, effect sizes, degrees of freedom and P value noted<br>*Give P values as exact values whenever suitable.*
☒	☐	For Bayesian analysis, information on the choice of priors and Markov chain Monte Carlo settings
☒	☐	For hierarchical and complex designs, identification of the appropriate level for tests and full reporting of outcomes
☒	☐	Estimates of effect sizes (e.g. Cohen's d, Pearson's r), indicating how they were calculated

Our web collection on statistics for biologists contains articles on many of the points above.

Software and code

Policy information about availability of computer code

Data collection	gnomAD r2.0.2. was used to measure allele frequency.<br>STRING v10.5 database was used for the base network model.<br>Discovery studio 2018 software (Dassault Systèmes BIOVIA, Discovery Studio Modeling Environment, Release 2018, San Diego: Dassault Systèmes, 2018.) was used for protein structure modeling study.<br>Digitizer 1550B and pClamp10 software were used for measurements in electrophysiology study.
Data analysis	Image J was used to analyze immunostaining data.<br>GraphPad Prism7 was used for graphing and statistical analysis.<br>Cas-OFFinder and Cas-Analyzer (http://www.rgenome.net/) were used for CRISPR target design and assessment of deep sequencing data.

For manuscripts utilizing custom algorithms or software that are central to the research but not yet described in published literature, software must be made available to editors/reviewers. We strongly encourage code deposition in a community repository (e.g. GitHub). See the Nature Research guidelines for submitting code & software for further information.

Data

Policy information about availability of data

All manuscripts must include a data availability statement. This statement should provide the following information, where applicable:

- Accession codes, unique identifiers, or web links for publicly available datasets
- A list of figures that have associated raw data
- A description of any restrictions on data availability

Targeted deep sequencing data have been deposited in the NCBI Sequence Read Archive database (SRA; https://www.ncbi.nlm.nih.gov/sra) under accession number PRJNA531568.

Field-specific reporting

Please select the one below that is the best fit for your research. If you are not sure, read the appropriate sections before making your selection.

☒ Life sciences ☐ Behavioural & social sciences ☐ Ecological, evolutionary & environmental sciences

For a reference copy of the document with all sections, see nature.com/documents/nr-reporting-summary-flat.pdf

Life sciences study design

All studies must disclose on these points even when the disclosure is negative.

Sample size	This research showed a personalized medicine approach, therefore we have studied with a limited number of one patient. From the iPS cells derived from the patient's fibroblast, we generated 2 isogenic ATP7A gene corrected iPS cell lines.
Data exclusions	All prepared samples were analyzed and all data were included
Replication	In experiments using NPC and MN, all experiments were biologically reproduced across 2 independent differentiations. In experiments using iNSC, all experiments were reproduced at least 4 times. All attempts at replication were successful.
Randomization	For neuron differentiation efficiencies and viability analysis, cell was immunostained with antibodies and photos were randomly taken. For electrophysiology, single cells were randomly selected for measurement.
Blinding	Neurons were selected by an individual who was blinded in neuron differentiation efficiencies and viabilities. All quantifications were performed by two researchers blinded to the identity of each sample.

Reporting for specific materials, systems and methods

We require information from authors about some types of materials, experimental systems and methods used in many studies. Here, indicate whether each material, system or method listed is relevant to your study. If you are not sure if a list item applies to your research, read the appropriate section before selecting a response.

Materials & experimental systems

n/a	Involved in the study
☐	☒ Antibodies
☐	☒ Eukaryotic cell lines
☒	☐ Palaeontology
☒	☐ Animals and other organisms
☐	☒ Human research participants
☒	☐ Clinical data

Methods

n/a	Involved in the study
☒	☐ ChIP-seq
☒	☐ Flow cytometry
☒	☐ MRI-based neuroimaging

Antibodies

Antibodies used	The following antibodies were used in this manuscript:MAP2 (Abcam), cleaved caspase-3 (Cell Signaling Technology), Ki67 (ZYMED, Abcam), PAX6 (DSHB), Sox2 (R&D Systems), Nestin (Santa Cruz), PLZF and N-CAD (Millipore); 4′, 6-diamidino-2-phenylindole (DAPI) (Santa Cruz Biotechnology) and Hoechst (Millipore). All antibodies were used for immunocytochemical analysis.
Validation	These antibodies were validated by the vendor or published studies using immunocytochemistry analyis.

Eukaryotic cell lines

Policy information about cell lines

Cell line source(s)	Reprogrammed iPS cells from a patient's fibroblast and a control line were provided by Zachary T. McEachin
Authentication	The fibroblast have not been authenticated by our lab independently.
Mycoplasma contamination	All cell lines were tested and confirmed to be negative for mycoplasma contamination.
Commonly misidentified lines (See ICLAC register)	No commonly misidentified cell lines were used.

Human research participants

Policy information about studies involving human research participants

Population characteristics	Sample name, Mutated gene, Disease, Age of onset, Gender<br>Control, None, None, None, M<br>ATP7A-M1311V, ATP7A, ALS, 60, M
Recruitment	This study was conducted for one patient who was diagnosed with ALS by a neurosurgeon, Nicholas M. Boulis. He agreed to use his tissue for a basic research.
Ethics oversight	Yonsei University

Note that full information on the approval of the study protocol must also be provided in the manuscript.
